# Microtremor data and HVSR method of geothermal manifestation of Mt. Telomoyo, Central Java, Indonesia

**DOI:** 10.1016/j.dib.2023.109721

**Published:** 2023-10-24

**Authors:** Gatot Yuliyanto, Tony Yulianto

**Affiliations:** a Department of Physics, Faculty of Science and Mathematics, Diponegoro University, Prof. H. Soedarto, SH Street, Tembalang District, Semarang City, Central Jawa, Indonesia; b Research Cluster For Geothermal, Faculty of Science and Mathematics, Diponegoro University, Prof. H. Soedarto, SH Street, Tembalang District, Semarang City, Central Jawa, Indonesia

**Keywords:** Seismic, Density, v_p_, v_s_, Umbul Temple, Dukuh Temple, Pakis Dadu

## Abstract

The data presented contains a collection of microtremor measurement data regarding the structures and fluid flow of the Mt. Telomoyo's geothermal manifestation, Indonesia. The data are primary data taken using 3 unit single-station microtremor devices with a total of 60 acquisition stations in The Umbul Temple, Dukuh Temple and Pakis Dadu hot spring manifestation area. Data was obtained using 3-component digital seismograph VHL PS-2B series and recorded with a data logger type GL 240 with a duration of 10 min. This data can then be used in the analysis of the Horizontal to Vertical Spectral Ratio (HVSR) which will later produce the HV curve. The compressional wave velocity v_p_, shear wave velocity v_s_, density, and thickness profiles of each data were obtained from carrying out the inversion process using Dinver.

Specifications Table

**Table:** 

Subject	Geophysics, Physics, Earth Sciences
Specific subject area	Near-surface geophysics, Tectonic, Geomorphology, Geothermal
Data format	Raw data (.CSV), image(.jpg), table (.xlsx) and analyzed
Type of data	Excell & Fig.
Data collection	Data acquisition was carried out by measuring microtremor signals using 3 unit 3-component digital seismograph VHL PS 2B serie and recorded with a data logger type GL 240. Field conditions and coordinates were recorded during raw data measurement. The potential for movement or disturbance around the seismogram is also recorded as noise in the data. The seismometer must be checked first so that it is integrated with the data logger. Placement of the seismometer must be in an area that has stable soil, cleared of gravel, grass and roots. Make sure the bubbles on the seismometer are right in the middle of the bull's eye level and the direction of the seismogram is pointing north. In this paper, 10 min of microtremor data is considered sufficient to represent each location.
Data source location	City/Town/Region: Mt. Telomoyo geothermal manifestation, Central Java Country: Indonesia Latitude and longitude samples/data −73105451, 1104271151
Data accessibility	https://data.mendeley.com/datasets/s6dhfd5m3c/1

## 1 Value of the Data

• Microtremor data can be used to improve further testing on other geothermal manifestation cases
• The microtremor data set can be used as a comparison and linked to other geophysical measurement tools or methods
• The data allows for analysis of lithology and subsurface structures, such as stratification within geological units, and fluid flow of geothermal manifestation
• Raw data can be reprocessed so that it can display 3D images of conceptual model of Mt Telomoyo geothermal manifestation
• Data processing can be carried out up to data inversion to determine the value of soil layer depth, vp, vs and density.

## 2 Data Description

**Fig. 1:**
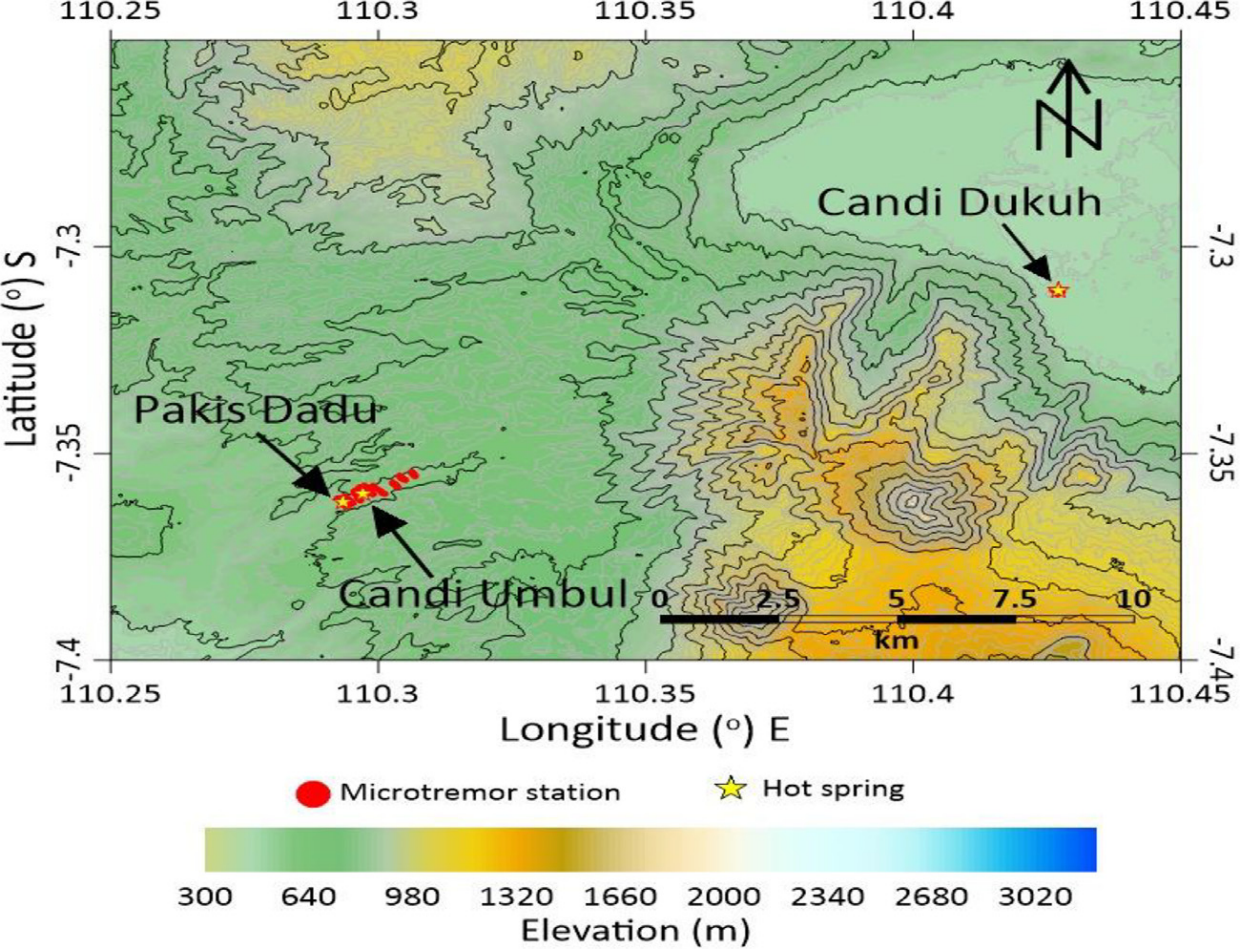
Acquisition of microtremors data.

**Fig. 2:**
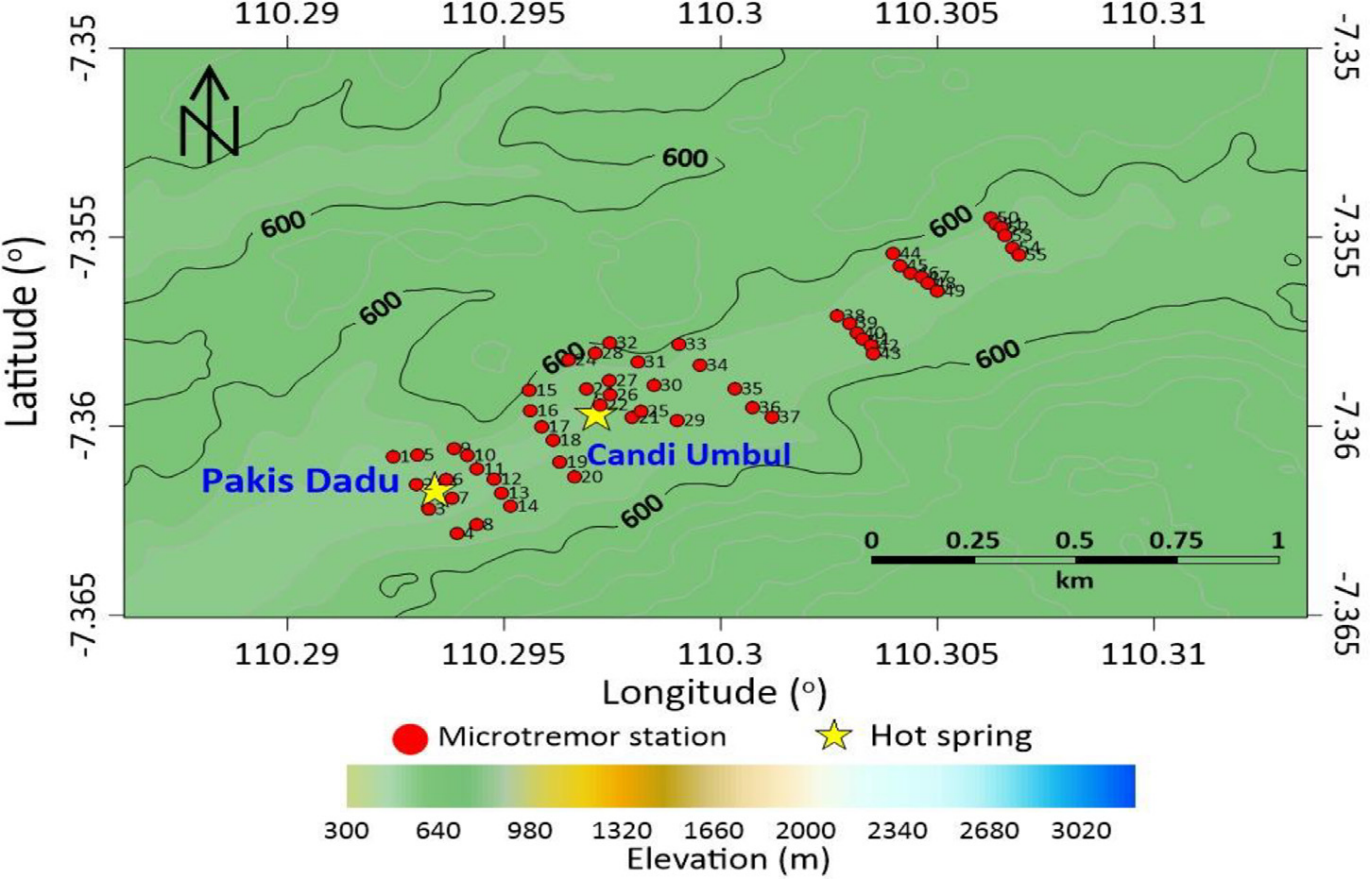
Acquisition of microtremors data in The Umbul Temple and Pakis Dadu.

**Fig. 3:**
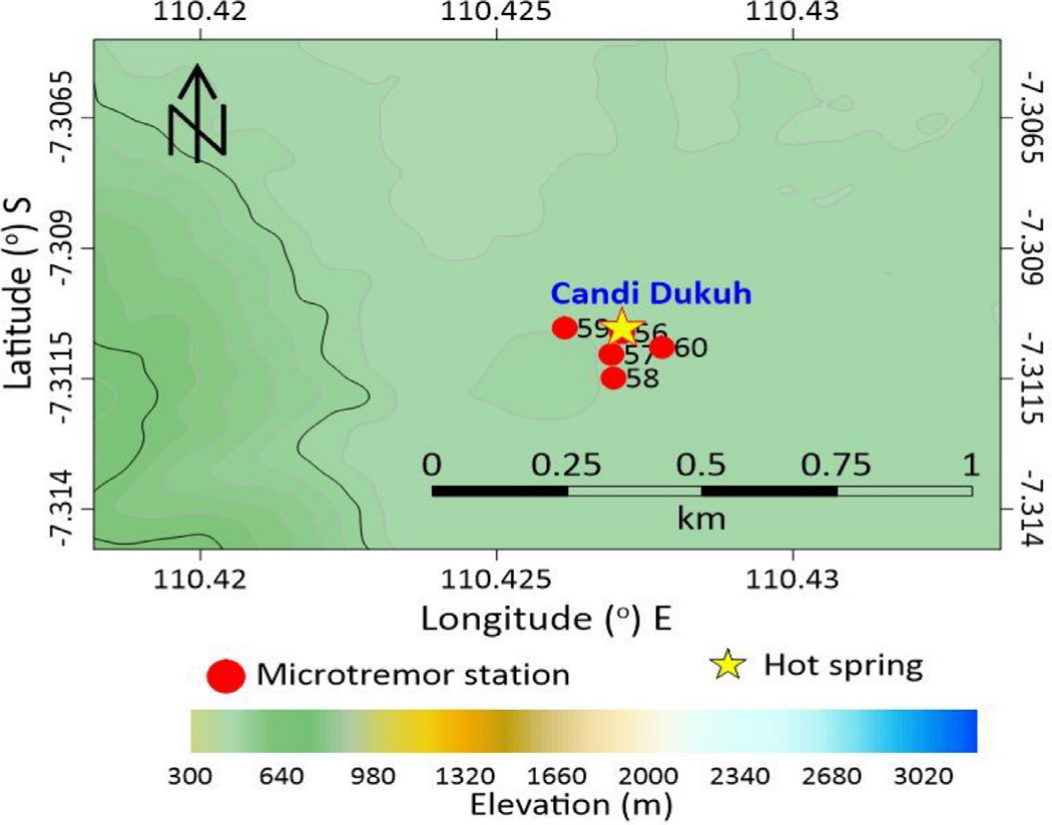
Acquisition of microtremors data in The Dukuh Temple.

Mount Telomoyo is one of several mountains that have potential as a source of geothermal energy. The outflow zone on Mount Telomoyo is in the area around the manifestations of Dukuh Temple, Umbul Temple and Pakis Dadu in the form of hot springs. The upflow zone of the Telomoyo geothermal system is formed within the Telomoyo complex caldera [1,2]. Fig. 1, Fig. 2, Fig. 3 shows the location of microtremor data collection with a total of 60 stations around the geothermal manifestation area of Mount Telomoyo in 2D form. The vertical side in Fig. 1, Fig. 2, Fig. 3 depicts the latitude and the horizontal side depicts the longitude of the research station location. Fig. 1 depicts the research location in general and the star images shown in Figs. 1–3 depict the location of the hot springs from Dukuh Temple, Umbul Temple and Pakis Dadu. Fig. 2 shows the research location at Umbul Temple and Pakis Dadu with a total of 55 stations, while Fig. 3 shows the research location at Dukuh Temple with a total of 5 stations. The elevation of each data stations varies greatly, which is around 558–760 m above sea level. Location data regarding the location of each measurement station in longitude, latitude and altitude are written in an attachment in the .xlsx format which can be opened using Ms. excell.

This research in the Mount Telomoyo area was carried out using a microtremor device consisting of a data logger and a 3-component digital seismograph VHL PS 2B, global positioning system (GPS), and compass to determine the North direction from data collection [3]. Software that can be used later for data processing is Microsoft Excel, Notepad, Notepad++, Geopsy, and Dinver for microtremor data, Surfer 13 for modeling subsurface conditions of geothermal manifestation of the Mount Telomoyo area [4]. The raw microtremor data obtained is in the form of .CSV which can be opened in the Microsoft Excel application. The data in the .CSV depicts the microtremor signal received by the seismograph and recorded by the data logger as seen in Fig. 2.

**Fig. 4:**
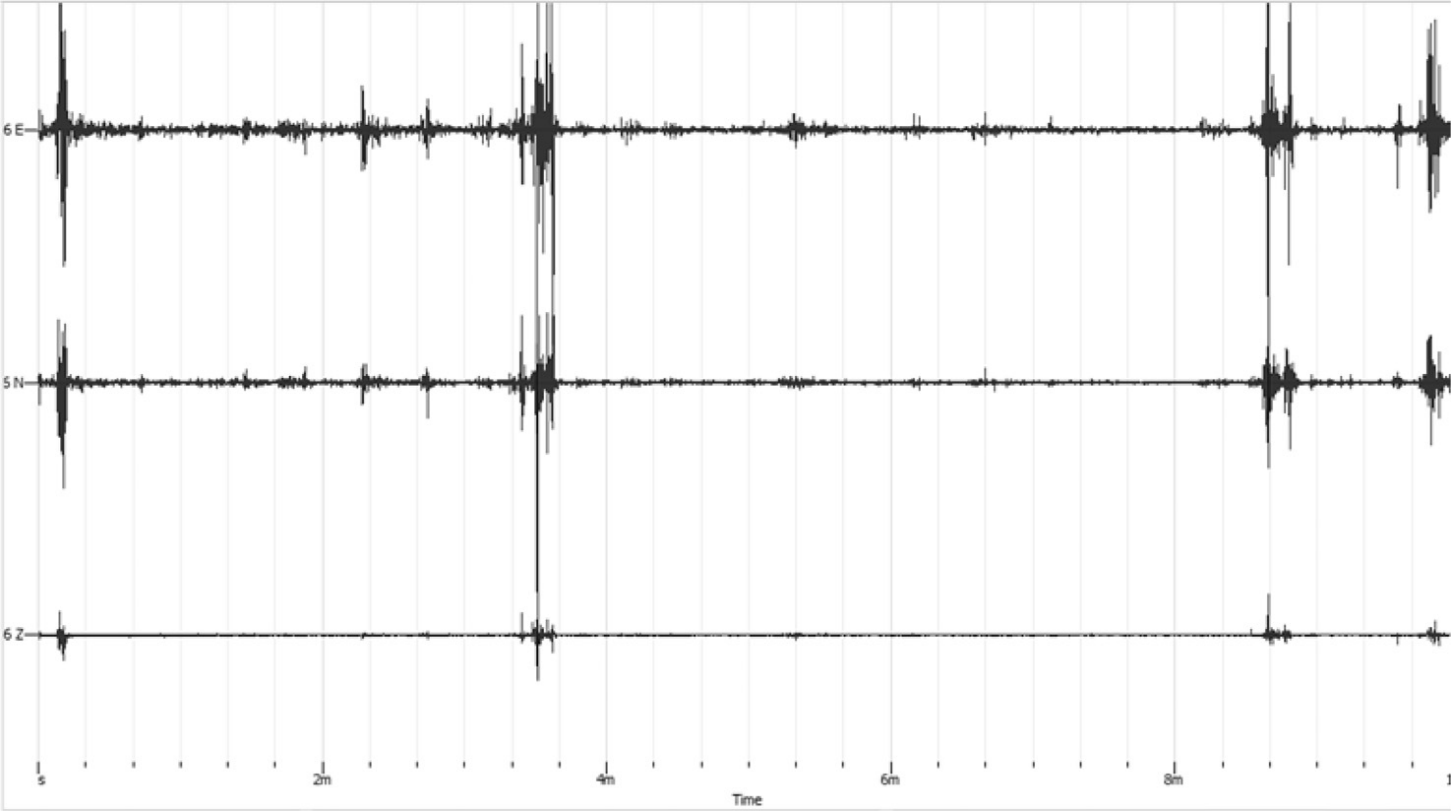
The HVSR Raw data at station 6.

The 3 components of microtremor consist of 2 horizontal components namely East-West & North-South and 1 vertical component. Fig. 4 shows the raw microtremor data opened in geopsy software. The top sections show the horizontal East-West component, he middle is the horizontal North-South component, and then the bottom sections show the vertical component. The vertical side of the whole of Fig. 4 shows the value of the microtremor signal while the horizontal side shows the measurement time. Processing with geopsy software will later provide dominant frequency values, amplification factors and HV curves. Then the hv curve can be inverted using Dinver to determine the value of vp, vs, density and layer thickness [5].

## 3 Experimental Design, Materials and Methods

### 3.1 HVSR Methods

The HVSR method is a geophysical method that can compare the spectrum of the horizontal component to the vertical component of microtremor waves. The processing method with HVSR provides information about the dominant frequency, hv curve and amplification value at the subsurface [6]. The amplification value of an area can be obtained from the peak spectrum of the HVSR curve from the results of the microtremor test in that area. The frequency and period values obtained from the HVSR curve can be correlated with the thickness of the sediment layer [7]. The HVSR method can be used in geothermal exploration. Some of the parameters that can be used in this analysis are the vs, vp/vs [8,9] and Poisson's ratio values [10,11]. Based on the property values of shear waves that are difficult to pass through fluid, it can be known about the structure, lithology or flow pattern of geothermal energy [12].

### 3.2 Data Acquisition

Data collection begins with designing a microtremor measurement survey around Mount Telomoyo using Google Earth Pro 7. Data collection at each station begins with checking the equipment and installing the cable that connects the geophone and the data logger. The next process is to determine the North direction using a geological compass to determine the Y-axis. Then adjust the bubble on the seismometer to be right in the middle of the bull's eye level. After activating the data logger and booting, vibration recording is complete. Data measurements at each station were carried out for 10 min and continued by storing data into the data logger [5]. The data logger will store each measurement station and generate data in .CSV format.

## Limitations

None.

## Ethics Statement

This work does not involve human subjects, animal experiments, or any data collected from social media platforms.

## CRediT authorship contribution statement

**Gatot Yuliyanto:** Conceptualization, Methodology, Formal analysis, Supervision, Investigation, Writing – original draft, Writing – review & editing. **Tony Yulianto:** Validation, Software, Formal analysis, Investigation, Data curation.

## Data Availability

Microtremor Research Data for Geothermal Manifestation MT. Telomoyo (Original data) (Mendeley Data)
